# Tracking a mass mortality outbreak of pen shell *Pinna nobilis* populations: A collaborative effort of scientists and citizens

**DOI:** 10.1038/s41598-019-49808-4

**Published:** 2019-09-16

**Authors:** Miguel Cabanellas-Reboredo, Maite Vázquez-Luis, Baptiste Mourre, Elvira Álvarez, Salud Deudero, Ángel Amores, Piero Addis, Enric Ballesteros, Agustín Barrajón, Stefania Coppa, José Rafael García-March, Salvatore Giacobbe, Francisca Giménez Casalduero, Louis Hadjioannou, Santiago V. Jiménez-Gutiérrez, Stelios Katsanevakis, Diego Kersting, Vesna Mačić, Borut Mavrič, Francesco Paolo Patti, Serge Planes, Patricia Prado, Jordi Sánchez, José Tena-Medialdea, Jean de Vaugelas, Nardo Vicente, Fatima Zohra Belkhamssa, Ivan Zupan, Iris E. Hendriks

**Affiliations:** 1 Oceanography and Global Change Department, Mediterranean Institute for Advanced Studies (CSIC-UIB), Esporles, Mallorca Spain; 2 Instituto Español de Oceanografía (IEO), Centro Oceanográfico de Baleares, Muelle de Poniente s/n, 07015 Palma de Mallorca, Spain; 3 Balearic Islands Coastal Observing and Forecasting System (SOCIB), Palma, Spain; 4 University of Cagliari, Department of Environmental and Life Science, Via Fiorelli 1, 09126 Cagliari, Italy; 5 Centre d’Estudis Avançats de Blanes-CSIC, 17300 Blanes, Girona Spain; 6 Agencia de Medio Ambiente y Agua, Consejería de Medio Ambiente y Ordenación del Territorio, Junta de Andalucía, Spain; 7 Consiglio Nazionale delle Ricerche-Istituto per lo studio degli impatti Antropici e Sostenibilità in ambiente marino (CNR - IAS), Oristano, Italy; 8 IMEDMAR-UCV, Institute of Environment and Marine Science Research, Universidad Católica de Valencia SVM, Calpe, Alicante Spain; 9 Department of Chemical, Biological, Pharmaceutical and Environmental Sciences (CHIBIOFARAM), University of Messina, Viale F. Stagno d’Alcontres 31, 98166 Messina, Italy; 10 Marine Science and applied Biology Department, University of Alicante, Alicante, Spain; 11 Enalia Physis Environmental Research Centre, Acropoleos 2, 2101 Aglantzia, Nicosia Cyprus; 12 Instituto de Ecología Litoral, El Campello, Alicante Spain; 13 University of the Aegean, Department of Marine Sciences, Mytilene, Greece; 14 Working Group on Geobiology and Anthropocene Research, Institute of Geological Sciences, Freie Universität Berlin, 12249 Berlin, Germany; 15 Institute of marine biology, University of Montenegro, Kotor, Montenegro; 16 National Institute of Biology, Marine Biology Station, Piran, Slovenia; 17 Integrative Marine Ecology, Stazione Zoologica Anton Dohrn, Villa Comunale, 80121 Naples, Italy; 18 PSL Research University, EPHE-UPVD-CNRS, USR 3278 CRIOBE, Université de Perpignan, 52 Avenue Paul Alduy, 66860 Perpignan, France; 19 Laboratoire d’Excellence CORAIL, BP 1013, 98729 Papetoai, Moorea French Polynesia; 20 IRTA-Marine and Continental Waters. Ctra. Poble Nou Km 5.5, 43540 Sant Carles de la Ràpita Tarragona, Spain; 21 SUBMON: Divulgació, Estudi i Conservació de l’Entorn Natural, Barcelona, Spain; 22 Université Côte d’Azur, CNRS, UMR 7035 ECOSEAS, Parc Valrose 28, Avenue Valrose, 06108 Nice, France; 23 IMBE (Institut Méditerranéen de Biodiversité et d’Ecologie marine et continentale), Aix-Marseille Univ., Avignon Univ., CNRS, IRD; and Institut Océanographique Paul Ricard, Ile des Embiez, 83140-Six Fours les Plages, France, Ile des Embiez, France; 24 Laboratory of Protection, Valorisation and Management of Marine and Littoral Resources & Molecular Systematics, Department of Marine Sciences and Aquaculture, Faculty of Natural Science and Life, Abdelhamid Ibn Badis University of Mostaganem, Mostaganem, 27000 PO Box 300, Algeria; 25 University of Zadar, Department of Ecology, Agronomy and Aquaculture, Zadar, Croatia

**Keywords:** Conservation biology, Conservation biology, Ecological epidemiology, Ecological epidemiology

## Abstract

A mass mortality event is devastating the populations of the endemic bivalve *Pinna nobilis* in the Mediterranean Sea from early autumn 2016. A newly described Haplosporidian endoparasite (*Haplosporidium pinnae*) is the most probable cause of this ecological catastrophe placing one of the largest bivalves of the world on the brink of extinction. As a pivotal step towards *Pinna nobilis* conservation, this contribution combines scientists and citizens’ data to address the fast- and vast-dispersion and prevalence outbreaks of the pathogen. Therefore, the potential role of currents on parasite expansion was addressed by means of drift simulations of virtual particles in a high-resolution regional currents model. A generalized additive model was implemented to test if environmental factors could modulate the infection of *Pinna nobilis* populations. The results strongly suggest that the parasite has probably dispersed regionally by surface currents, and that the disease expression seems to be closely related to temperatures above 13.5 °C and to a salinity range between 36.5–39.7 psu. The most likely spread of the disease along the Mediterranean basin associated with scattered survival spots and very few survivors (potentially resistant individuals), point to a challenging scenario for conservation of the emblematic *Pinna nobilis*, which will require fast and strategic management measures and should make use of the essential role citizen science projects can play.

## Introduction

The pen shell *Pinna nobilis* (Linnaeus, 1758), is the largest bivalve of the Mediterranean Sea where it is endemic; and with its size of up to 1.2 m height ranks as one of the largest worldwide^1–3^. This species grows on soft-bottom coastal areas and mainly inhabits seagrass meadows (dominated by *Posidonia oceanica* and *Cymodocea nodosa*), but also occasionally thrives on unvegetated bottoms, maërl beds, or among boulders^2,4–6^. In favourable conditions the lifespan of *P. nobilis* is up to 50 years^7^ and the species develops a key ecological role. Pen shells filter water and retain large amounts of organic matter from suspended detritus^8^, contributing to water clarity. In addition, its shell provides a hard-surface within a soft-bottom ecosystem, which is colonized by many different benthic species^9–11^, and at high densities can create high-diversity biogenic reefs^12^. It also plays a key role in the trophic web, serving as prey of other species (e.g., *Octopus vulgaris*^13,14^) and host of symbionts like the crustaceans *Pontonia pinnophylax* and *Nepinnotheres pinnotheres*^15^.

Several anthropogenic pressures such as pollution^16,17^, anchoring^18,19^, harvesting^20^, habitat degradation^21,22^ and environmental threats like for example global warming, which induces a decrease in juveniles’ survival rate^23^ have contributed to accelerate the decline of pen shell populations in the last century, making this emblematic species one of the most important bio-indicators of ecosystem status in the Mediterranean basin. This decline has in turn led to list the pen shell as an endangered and protected species under Annex IV of the Habitats Directive (European Council Directive 92/43/EEC), Annex II of the Barcelona Convention, and national legislation in most Mediterranean countries.

Recently, pen shell’s population health in Spain has plummeted, causing concern and a status change from “Vulnerable” category to “Critically Endangered” with a serious extinction risk (Orden TEC/1078/2018). This is due to a Mass Mortality Event (MME^24,25^) that affected *P. nobilis* populations of the south-western Mediterranean Sea starting in early autumn 2016 with extremely high mortality levels (reaching up to 100% at monitored populations^26^). Thereafter, severe mortality was also observed in pen shell populations from the north-western Mediterranean (Italian and French coasts^27^) and as far as the Aegean sea^28^. As in two regions of Italy (Campania and Sicily) a mycobacterial disease has recently been associated with the mortality episodes of *P. nobilis*^29^, the role of a *Mycobacterium* sp. in a complex disease pathogenesis cannot be discarded. However, also in Italy^30^ there is compelling evidence that the main cause of this mortality wave is the protozoan *Haplosporidium pinnae*, a new species genetically related to the group of Haplosporidian parasites^27^. Different life stages of this parasite have been detected in diagnosed sick pen shells. The sporulation stage of the protozoa has been detected in the digestive gland, while the uninucleate and the less frequent plasmodial stages have been found in the connective tissue and the gut epithelium. This type of infection triggers a heavy inflammatory response with harmful physiological responses for the host (e.g., precluding the digestion process), finally causing the death of the animal^27,31^.

Haplosporidian endoparasites have been the cause of mortality events in other bivalve populations. They have been specifically well-studied in cases of molluscs of commercial interest, most likely due to the socio-economic repercussions. The best studied members of the group are *Haplosporidium nelsoni* and *Bonamia ostreae* which cause die-backs in the oysters *Crassostrea virginica* (Atlantic coasts of USA^32,33^) and *Ostrea edulis* (European coasts^34,35^), respectively. Several studies suggest a key role of the environment affecting the life-cycle of these parasites^32,36,37^. The disease prevalence and intensity shows seasonal patterns; for example, a peak of prevalence at higher summer temperatures and salinity was observed for *H. nelsoni*^38,39^ and *Bonamia exitiosa*^40,41^. While *B. ostreae* shows the same pattern for salinity (prevalence decreases at lower salinities), in contrast, the parasite outside its host showed higher survival and esterase activities at 4 °C and 15 °C than at 25 °C^42,43^. Little is known about the environmental dispersal of Haplosporidians although oceanic currents have been suggested as a potential factor driving the expansion of these parasites^42,44^. The quick northward expansion of the disease along the Iberian peninsula during the present outbreak^26^, with early outbreaks in the Central Mediterranean^28^, agrees with the hypothesis of a pivotal role of currents in disease dispersal at regional but also basin-wide scales. The occurrence of healthy *P. nobilis* populations in specific areas (e.g., Alfacs Bay in the Ebro Delta, with low salinity caused by freshwater discharges) points towards a relation between environmental factors such as salinity and/or temperature on disease prevalence or survival of *H. pinnae*.

However, the study of rapid and vast pathogen dispersal (e.g., invasive diseases) as well as the relationship with environmental parameters is often constrained by the scale^45^ and time-period needed for observations. The problem of intense sampling effort can be resolved with networks of citizen and scientists, which can multiply the spatio-temporal sampling effort without the economic and logistic constraints of scientific sampling^46^. Here, we have combined observations of scientists and citizens to track the mass mortality outbreak caused by the new species-specific parasite *H. pinnae*^27^ in order to test if dispersal speed is coherent with local current patterns and to investigate if there are environmental factors aiding the infection of pen shell populations by the pathogen. This could provide an indication of further future dispersal of the pathogen beyond the western Mediterranean basin and current infection areas and pin-point populations at risk in the near future.

## Results

**Figure 1:**
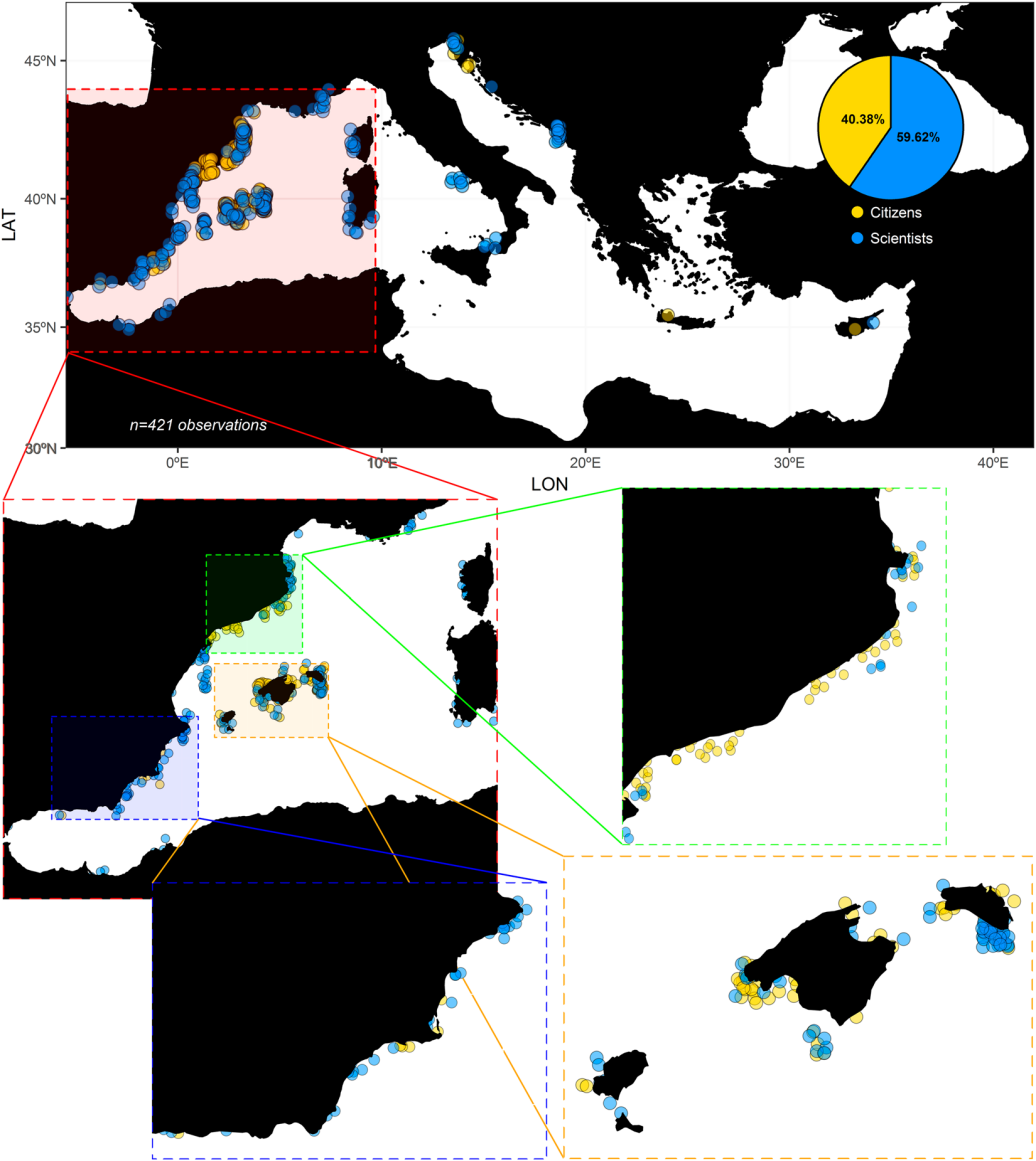
Observations of *P. nobilis* health conducted by citizens (yellow circles) *vs*. scientists (blue circles), with zoomed in areas where most observations were done.

**Figure 2:**
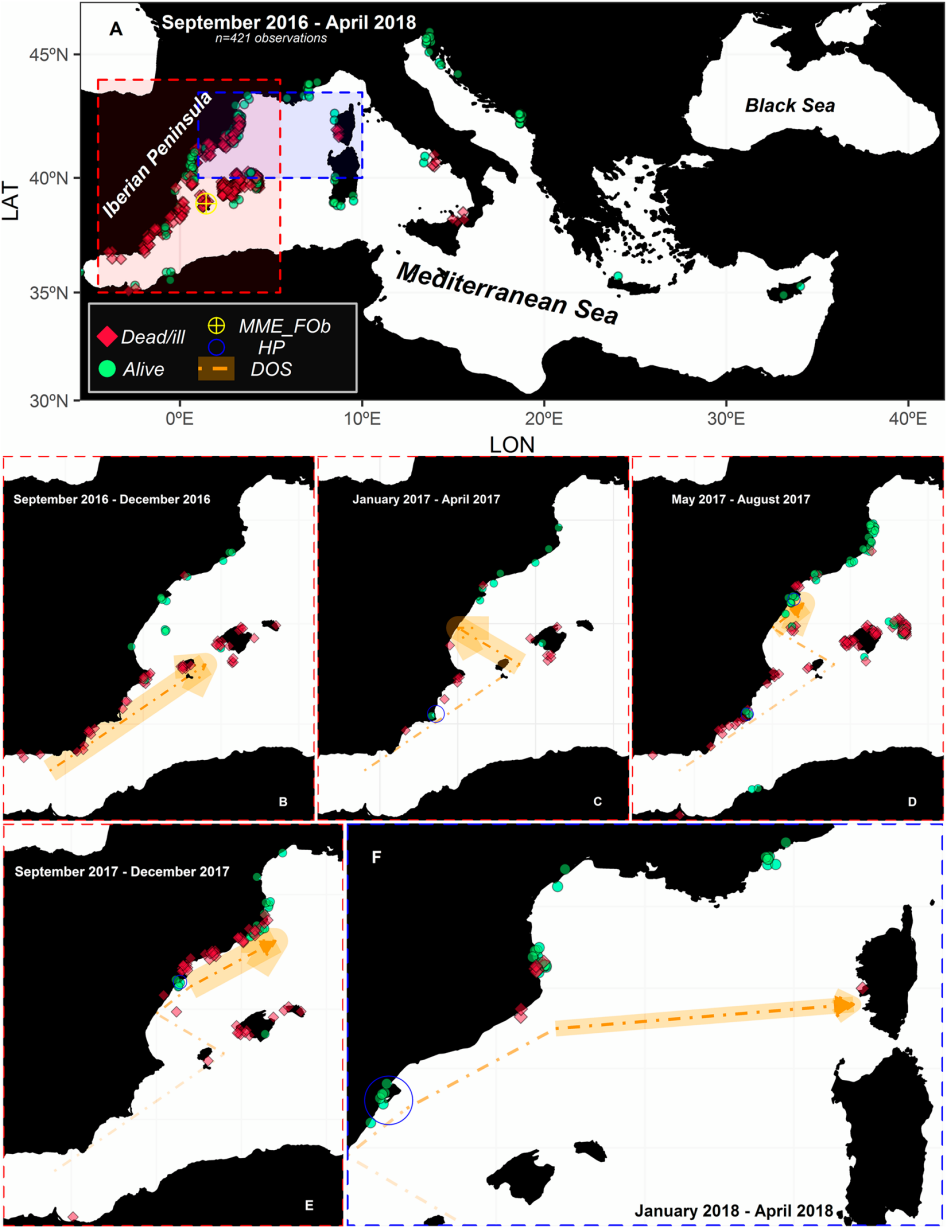
Disease observations based on the health status of *P. nobilis* (Dead/ill *vs*. alive, represented by red diamonds and green circles respectively) for the whole period from September 2016 to April 2018 (**A**). Spatio-temporal zooms of such observations are represented in panels B–F. Note that the crossed yellow point indicates the zone where the mortality was observed for the first time (Mass Mortality Event First Observation; *MME_FOb*), blue empty circles surround Healthy Populations (*HP*) and orange arrows denote Disease Observations Sequence (DOS).

A total of 251 observations from September 2016 to April 2018 reported by the Mediterranean scientific network were combined with the 170 observations by citizens (June 2017–April 2018; ~26 monthly observations) to yield a total of 421 observations (40.38 and 59.62% reported by citizens *vs*. scientists respectively; Fig. 1 and Table A in Supplementary Material). Initially 289 observations were reported from the sea-watchers’ platform, which were scrutinized by expert scientists to filter out possible confusions between congeneric species *P. nobilis* and *P. rudis* as well as other kind of errors in the reports by sea-watchers. The spatio-temporal representation of this combined data set (Fig. 2) points to the southern part of the Iberian Peninsula as the first region where the MME was detected (Fig. 2A), while the Balearic Islands were the second area affected by this severe mortality in a relatively short time (Fig. 2B). Later, and after a relatively long period of time (8 months), the signs of the disease were sequentially detected on a northward trajectory along the Valencian coast (Fig. 2C,D). At the end of 2017, the MME was observed on the Catalan coast and beyond Spanish waters in some sites around Corsica (Fig. 2E). During the first months of 2018, mortality was observed at specific sites along the Italian coast (Fig. 2F). However, specific localities in the Mediterranean Sea still harboured *P. nobilis* populations in good condition for the duration of this mortality wave (from September 2016 to April 2018). For example, no signs of the disease have been detected at the Mar Menor coastal lagoon and Ebro Delta areas (highlighted with blue circles in Fig. 2C,F), despite the fact that there was 100% mortality in the surrounding areas. Similarly, in the entire Central and Eastern Mediterranean *P. nobilis* populations remained unaffected until April 2018 (Figs 1A,B and 2A in Supplementary Material).

**Figure 3:**
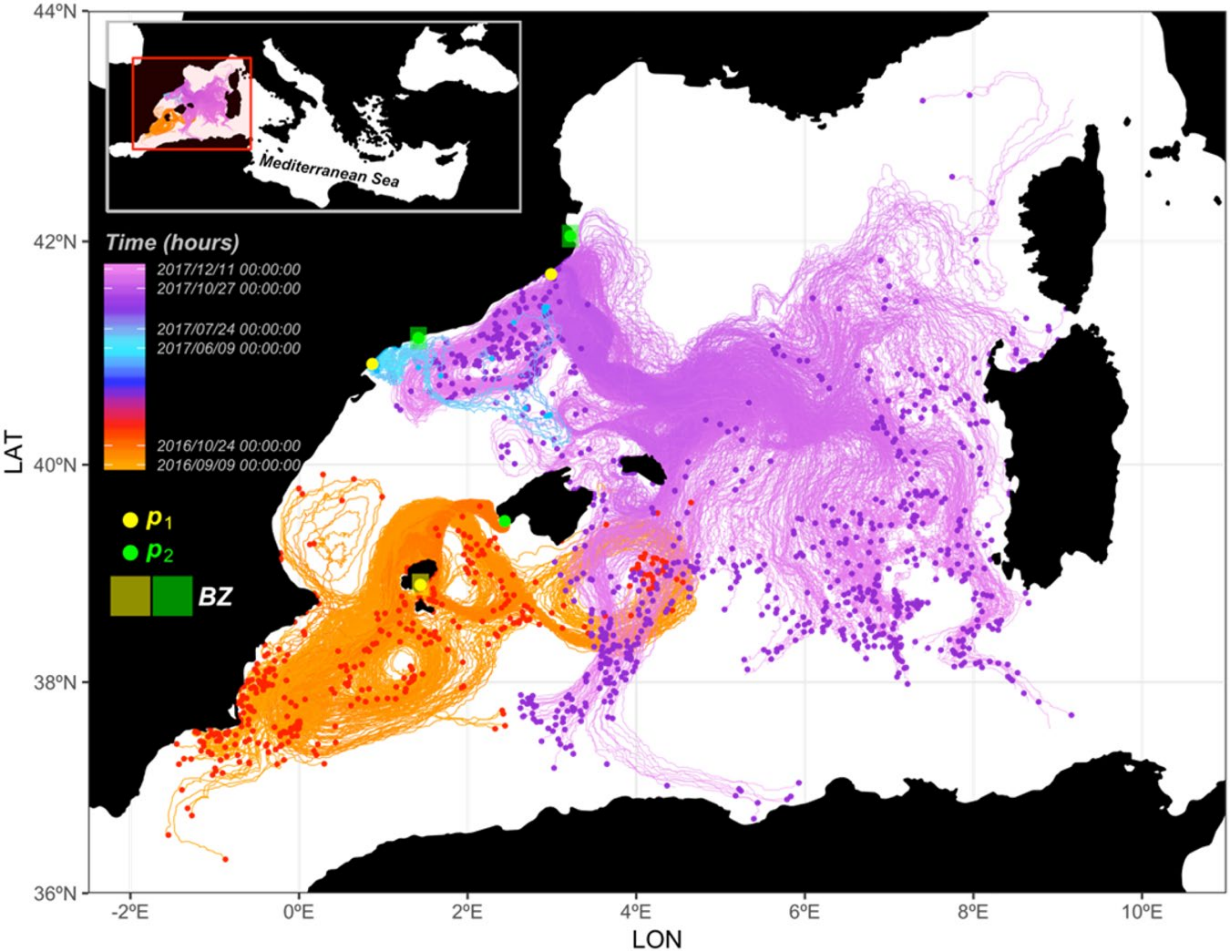
Drift simulations: backward simulations in the Balearic Islands area (orange path), forward simulations at L’Ametlla de Mar area (blue path) and, forward simulations at the north Catalonia area (purple path). Note that points at the end of the paths indicate the particle final position. Simulations were inferred between point 1 (*p*_1_; infected point) and point 2 (*p*_2_; no signs of infection at the beginning of the simulation and infection at a known time interval of the simulation). The rectangle indicates 650 km^2^ buffer zone around a given point.

The model current simulations suggest that the parasite *Haplosporidium pinnae* has probably dispersed regionally by currents (mp4 animation in Supplementary Material and Fig. 3). The backward trajectories ending on the southern coast of Mallorca (*p*_2_ at Fig. 3) in October 2016 indicate a possible origin along the coast of Alicante and Murcia, which is in agreement with the observations of the disease (orange trajectories at Fig. 3). Moreover, the transit time is consistent with the observations, showing only a 1-day difference between the average arrival time at *p*_2_ and the observed date (average time; Table 1). A similar agreement is found in the second simulation using forward trajectories from the Ebro River area in June 2017 (blue trajectories in Fig. 3). A slow northward propagation is described in the model, with an average time of arrival of the particles in the final point (*p*_2_) 3 days before the observed infection (Table 1). This agreement in both simulations is quite remarkable given the uncertainties inherently present in the model predictions. As mentioned in Section 4.2, the cluster approach provides a way to take into account these uncertainties. The backward simulation shows a wide range of possible departure times, with an average approximately at the observed date as the first particle left *p*_1_ 19 days before and last particle left *p*_1_ 18 days after the observed date (Table 1), providing insights into the range of possible scenarios for particle drifts given specific surface current conditions over a period of several weeks. The simulated departure dates for L’Ametlla de Mar are more skewed, as the first particle arrives to *p*_2_ 19 days before and last particle left *p*_2_ 10 days after the observed infection date (Table 1). The third simulation providing forward trajectories from a location along the Catalan coast in December 2017 (purple trajectories at Fig. 3) indicates an overall south-eastward dispersion towards Mallorca, Menorca, Corsica and Sardinia islands, and the Algero-Provençal basin. Some of the particles still reached the area of observed infection, with an average arrival 26 days before the observed infection date. The last particle reaching *p*_2_ arrived only 3 days before the observed infection (Table 1). Even if the agreement is not as good as in the two previous cases, these results still support the hypothesis of disease dispersion by surface currents given the uncertainties present in the model and observations.

**Table 1:** Observed *vs*. estimated dates for the arrival of particles subset at buffer areas in the three drift simulations conducted.

Simulation	Area	LonLat *p*_1_		LonLat *p*_2_		*Omme*_*f*_	*AEt*_*f*_	*FEt*_*f*_	*LEt*_*f*_
*Backward*	*Balearic Islands*	2.45°E	39.50°N	1.44°E	38.91°N	2016/09/28	2016/09/29	2016/09/09	2016/10/16
*Forward*	*L’Ametlla de Mar*	1.42°E	41.14°N	0.87°E	40.91°N	2017/07/14	2017/07/11	2017/06/25	2017/07/24
*Forward*	*North Catalonia*	2.99°E	41.71°N	3.22°E	42.05°N	2017/12/07	2017/11/11	2017/11/07	2017/12/04

Nomenclature:Longitude and latitude (LonLat) at point 1 (*p*_1_) and point 2 (*p*_2_); Observed date of MME (*Omme*_*f*_); Estimated average date (of particles subset) for the arrival of the particles at *p*_2_ (*AEt*_*f*_); First date for the arrival of the particles at *p*_2_ (*FAEt*_*f*_); Last date for the arrival of the particles at *p*_2_ (*LAEt*_*f*_).

**Figure 4:**
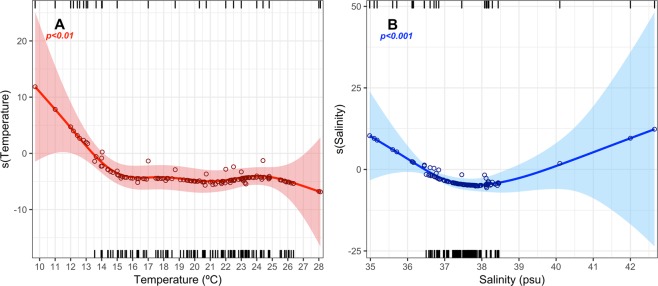
Partial smoothed effect of Temperature (**A**) and Salinity (**B**) on the disease expression (note that zero marks the shift on the response; >0 corresponds to higher probability of disease absence, while <0 to higher probability of disease expression). Fitted lines (solid line), 95% confidence intervals (shaded area) and partial residuals (hollow points) are shown for univariate effects. Top and bottom tick marks on *x*-axis represent values of the observed covariable for the absence and presence of signs of infection respectively.

After arrival to local sites, temperature and salinity appear to be decisive in the development of the disease (p < 0.01 and p < 0.001, respectively; 69.5% deviance explained) according to the GAM model. In fact, the disease expression (pen shell dead or ill) seems to be closely related to temperatures above 13.5 °C and with a salinity range between 36.5 and 39.7 psu (Fig. 4).

## Discussion

Assuming that the mass mortality events described in this study have been caused by the newly described parasite *H. pinnae*^27^, model simulations of particle drift dictated by regional surface currents suggest that parasite dispersion by surface currents could play an important role at a regional scale, as has been suggested before for other Haplosporidian species^44^. Despite the lack of resistant forms in *Bonamia* species (sporal stage or sporulation process have never been detected^27^), some studies support that *B. ostreae* and *B. exitiosa* spread via water currents and strong tide currents^42,47^ contributing to a rapid expansion but with a reduced spatial range (within a bay or in sites sharing commune water bodies but with reduced survival of the cells outside the host^47^). Such constricted dispersion does not seem likely for the pattern of parasite dispersion in this study, where the spores of *H. pinnae* would be dispersed over long distances for an extended time period (at least for a few weeks; Fig. 3 and mp4 animation in Supplementary Material). However, this hypothesis is compatible with Haplosporidians with a sporal stage, since though dispersal in most such parasite species (e.g., *H. nelsoni*) is poorly understood and remains purely speculative, several studies suggest that dispersion by currents is the most plausible dispersion mode^33,48^. Spores or at least sporulation of the pen shell parasite *H. pinnae* have been detected in the epithelium of digestive tubules^27,31^, and spore ornamentations, which may aid in flotation^49^, have been detected in other molluscan protozoan parasites^49^. Therefore, the presence of such resistant spores with attachments (e.g., long tape-like filaments attached to the spore wall) possibly providing buoyancy^27^ in the life-cycle of *H. pinnae*, suggests that this parasite could be readily dispersed by currents - the remarkable agreement obtained in this study between the arrival of virtual particles and observed infection at sites strongly supports this hypothesis. Alternatively, there could be a hypothetical involvement of planktonic species^50^ as an intermediate host, as suggested for *H. nelsoni* and *H. costale*^33,51^. The intrinsic uncertainty of the model linked to the turbulent nature of ocean flows and the existence of fine-scale littoral processes that remain unresolved in the model inevitably generate uncertainties in the computation of ocean trajectories. A major outcome of the modelling study was that some areas were successively infected with a time delay in reasonable agreement with the observations, which indicates that surface currents could act as a main driver for the transport of the parasite across the Mediterranean basin. Moreover, not only model simulations, but also the very limited knowledge of certain key stages of the parasite life cycle could be a potential uncertainty source. At the moment, the time-frame from parasite colonization of a given area and infection and virulence in the host is unknown; therefore pen shells might be infected for some time before showing signs of disease. This means that both scientists and sea-watchers only detect the infection when *P. nobilis* shows signs of disease (e.g., pale and withdrawn mantle, valves that do not close or do it very slowly), but the parasite could have the ability of infecting the pen shell some days, weeks or even months before. Moreover, an unknown time could elapse between the infection and the death of *P. nobilis*. These un-estimated and unknown time-steps could induce a potential bias causing the uncertainty and discrepancy between observations and predictions. Similarly, the origin of this parasite is speculative because of the limited information available for some areas such as the North-African coast. For instance, a die-off has been detected in certain counties along that coastline, but the timing of this event is completely unknown, which constricts our capacity to identify the exact source of the infection. Further uncertainty is also associated to the role and origin of the Mycobacteria species recently found in two regions of Italy and associated to mass mortality events of *P. nobilis*^29^. In otherwise healthy individuals, significant mortality (up to 82%) appears to be associated to the presence of *Vibrio mediterranei* and its degree of pathogenicity is currently under investigation (P. Prado, pers. communication).

The dynamics of Haplosporidian parasites is modulated by environmental conditions^44^ and therefore also the prevalence of *H. pinnae* after its arrival by currents at a given area is subject to the environmental conditions in such area. Based on our modelling results, the expression of disease seems to be related to temperatures above 13.5 °C and a salinity range between 36.5–39.7 psu, although the upper limit of the salinity should not be considered accurate due to the lack of such data (salinities above 38.5 psu) - monitoring further dispersal of the parasite in the eastern Mediterranean, where prevailing salinities are higher than in the western Mediterranean, will allow the accurate estimation of the upper salinity limit. A similar pattern has been also observed for other Haplosporidians parasites. For example, the congeneric species *H. nelsoni* is highly sensitive to temperature and salinity, which influence both its geographic distribution and seasonal infection patterns^32,36,37^. Specifically, field and *in vitro* experiments demonstrated that the ability of *H. nelsoni* to proliferate or survive decreases at lower salinities^38^ and its prevalence and virulence is affected seasonally by temperature oscillations; its prevalence decreases in the late winter, due to the exposure to low temperatures^39^. *B. exitiosa* shows a similar pattern of tolerance of environmental conditions^40,41^. However, *B. ostreae* showed higher survival outside its host and viability at lower temperatures (4–15 °C than at 25 °C), although the same positive relationship was observed regarding disease prevalence and salinity (higher prevalence at salinities >35 psu than <20 psu)^42^. These patterns suggest a species-specific tolerance to environmental conditions^44^, and could explain why, despite infection of the surrounding areas (with extinct populations), there are areas that shelter *P. nobilis* populations that are still in good condition. This is quite probably due to the salinity range outside the common Mediterranean salinity range of 36.7–39.5 psu^52^; this is the case, for example, of Alfacs Bay, strongly affected by discharges of the Ebro River^53,54^, and the Mar Menor coastal lagoon, a confined area with high salinity up to 40 psu^55^. However, it is important to remark that pen shells living in the Mar Menor lagoon or Alfacs Bay are vulnerable populations since changes in water conditions (currents, salinity and temperature) can still lead to the disappearance of those populations by a sudden exposure to the parasite or by increased virulence. Similarly, the observation of healthy *P. nobilis* populations at the northernmost Catalan Coasts and French Coasts in April 2018 (Fig. 2F) were probably related to the fact that the arrival of the parasite coincided with un-favourable low-winter temperatures (<13.5 °C^56^). In fact, by May 2018, when temperatures increased above 13.5 °C, mass mortalities were detected in these populations (pers. com. García-March).

In the last decades, the participation of citizens in science has experienced an exponential growth, probably due to the ecological awareness and the wide range of possibilities offered by the latest technologies (e.g, web portals, mobiles, apps to report live observations^57^). In this study, citizen science has played an essential role in the follow-up of a mass mortality event with a rapid expansion across an extensive area (western Mediterranean basin). Citizen science can overcome the logistics barriers of scientific monitoring, contributing with several observations at high spatial-temporal scales, as for example to track disease-carrying tiger mosquitoes^46^. However, citizen observations require a strict quality control for the reliability of data reported^58,59^. In our case, a scientific team was involved in the carefully validation of each citizen observation based on: (*i*) the experience of sea-watcher (e.g., scuba diving instructor *vs*. kayak fan, the first with a supposed better experience observing pen shells), (*ii*) complementary information (e.g., habitat type, with rocky bottoms the essential habitat for the congeneric species *P. rudis*) and, essentially, with (*iii*) photographic evidence (the most important and essential additional information for validation). In fact, more than half of the reported observations of healthy *P. nobilis* were removed (58.82%) after being carefully scrutinized by the scientific team. Most of such errant observations made reference to a very similar species; *P. rudis*. This congeneric species remains in good health conditions and seems to be resistant to the disease, with recent mortality rates according to its natural rates, and not higher than before the outbreak^6,60^. However it is difficult to discern between species due to anatomical similitudes^3^. When the technological problems of sharing observations are overcome, future efforts should be focused on minimizing the hard work of manually filtering citizens’ observations^58^. Notwithstanding the applicability of citizen observations, the best solution would appear to be the combination of citizen science with traditional scientific methods that combine tracking of mortality events in sentinel populations with detection of *H. pinnae*, allowing the two approaches to complement each other^46^. Therefore not only validation of layman observations, but also a coordinated, scientific monitoring program for the Mediterranean would be beneficial to follow the spread of mortality events.

Overall, our results point to a challenging scenario for the conservation of the emblematic *P. nobilis*. Most likely, currents will carry the parasite along the whole Mediterranean basin, and are already reaching the healthy populations of the central (e.g., Sardinia - June 2018; pers. com. Coppa) and eastern Mediterranean (e.g., Lesvos Island, Greece - August 2018^28^). In addition, the dispersal of the disease to distant localities could be assisted by vessels through their ballast waters, which constitutes one of the major vectors of primary and secondary introduction of alien species in the Mediterranean^61^. The extreme virulence of *H. pinnae*^26–28^ will probably devastate *P. nobilis* populations, except for populations confined in areas with environmental conditions beyond the tolerance limits of the parasite (constant low-temperatures and abnormally high or low salinity values) or the potential resistant individuals. The high genetic homogeneity of Mediterranean pen shell populations, with little differentiation between recently disappeared populations in more “open” waters and these scattered survival spots^62^, seriously compromises the recovery of the species as they likely share the same susceptibility for the parasite. Also, the inbreeding coefficient (F_IS_) was positive and significant at the western Mediterranean locations^62^. Inbreeding can greatly reduce the average individual fitness, and loss of genetic variability from random genetic drift can diminish future adaptability to a changing environment^63^. Despite the theoretical possibility that spots with surviving populations could act as a larval source for the spread of the species in larger areas^62^, this is only possible if they act as source populations, there is a favourable connection between these sites by local currents^64^, and recruits meet with parasite-free conditions in the localities of settlement. Another possible source for the recovery of populations might be the presence of surviving resistant individuals. However, this possibility seems to be compromised by several bottle-necks. Even if now and then there are sightings of healthy and maybe resistant individuals, they are extremely scattered, reducing the probability of reproduction among them (as male and female gametes are released to the water column and fertilization is external^21^), so relatively dense populations might be needed to assure successful fertilization. In the case of overcoming this reproductive handicap, if the scattered resistant individuals were able to produce some recruits, their natural mortality would be very high, because the species is extremely vulnerable to predators when young^6,14^. Furthermore, the pen shell’s slow growth-rate and dynamics (e.g., only individuals larger than 37 cm of shell length -at least 4 years old- released gametes in experimental conditions^65^) turn this species highly vulnerable to catastrophic events and predict a very slow recovery of the species even if recruits manage to establish in infected areas. Therefore, it is crucial to act while there are still specimens in healthy conditions, and to protect and monitor surviving individuals. As illegal harvesting of *P. nobilis* still occurs in many Mediterranean countries (e.g.^66^), better enforcement of the legislation for the protection of the species and actions for public awareness are needed to protect unaffected populations. The participation of citizens in protection campaigns and the monitoring of the status of populations all over the Mediterranean basin can play a key role.

## Material and Methods

### Data collection

We compiled data on mortality of pen shell populations starting from the first MME warning (28^th^ September of 2016 at Ibiza, Balearic Islands, Spain; crossed yellow point at Fig. 2A) to April 2018. Scientific data was compiled from: (*i*) data published in different scientific documents^26,67,68^ and (*ii*) scuba diving monitoring of pen shell populations conducted by most of the scientific groups involved in the study of the *P. nobilis* (expert network). In order to complement the scientific data with multiple and simultaneous observations at many sites, a module open to citizens was added to (project NACRAS; launched on 2^th^ of June of 2017) the website: http://www.observadoresdelmar.es (translated in English as “sea-watchers”). This platform allows citizens to report their observations about pen shell sightings and health state (alive, dead, or ill; the latter defined as a slow closing response of the valves or a pale and withdrawn mantle) with some complementary information (a spatio-temporal reference; date and GPS coordinates, temperature, salinity, depth, and habitat). After uploading an observation, the scientific team of the module curated each record based on sea-watcher experience, availability of complementary information and/or un uploaded photo, and frequently, through a dialogue with the sea-watcher.

From these sources, essential information like recent abnormal mortality, date, GPS coordinates, temperature, salinity, and depth were extracted for each observation to fit predictive models (see section Data analyses below). A trustworthy curated observation of one individual of a population reported as dead or ill under suspicious (not by predators, anchoring etc.) conditions was included in the model. Temperature and salinity data were extracted from the nearest available point of the database ‘*EN4.2.1: quality controlled subsurface ocean temperature and salinity profiles and objective analyses’* (downloaded from https://www.metoffice.gov.uk/hadobs/en4/ on April 30^th^ 2018^69^) except for sites where hydrographic values were carefully measured *in situ* by scientists (e.g., the Mar Menor coastal lagoon, the Alfacs Bay affected by River Ebro discharges). The *EN4.2.1* dataset contains monthly objective analysis of temperature and salinity with the corrections^69^ in a 1-degree grid and 42 different depths (from 5 m to 5000 m), spanning a period from 1900 to present.

### Data analyses

The potential role of the surface currents on the expansion of the disease was investigated simulating the drift of 1000 virtual particles (*Par*) in a high-resolution regional model of the western Mediterranean Sea, the WMOP model^70,71^. This model is developed and operated by SOCIB, the Balearic Islands Coastal Observing and Forecasting System. The WMOP model provides daily predictions of hydrodynamic conditions with a 2-km resolution from the Strait of Gibraltar to Corsica and Sardinia. It is a regional implementation of the Regional Ocean Modelling System (ROMS^72^), downscaling simulations from the Copernicus Marine Service Mediterranean model, and forced by the high resolution HIRLAM model from the Spanish Meteorological Agency. Details of the operational system can be found in^70,71^. An important aspect of this system is the multi-platform evaluation of the model predictions using available *in situ* and satellite observations, both in real-time and delayed modes^70,71^, including daily validation updates provided on SOCIB website. The surface currents predicted by the operational model in 2016 and 2017 were used to simulate the drift of virtual particles from locations and times associated with observations of infected *P. nobilis* during the following 45 days. The TRACMASS algorithm^73^ was used to generate the Lagrangian trajectories. A cluster of 1000 particles was seeded at each starting location, and advected by model surface currents plus a diffusive term adding a random displacement at each time step to account for model uncertainties and unresolved processes. Simulations were performed both in forward and backward modes to track the potential destination and provenance of the particles as described by the model surface current conditions. More concretely, simulations of currents were conducted in areas and within time-intervals (*t*_0_, … *t*_*n*_; time in days) where the arrival of the disease (final time; *t*_*f*_) at a given point (*p*_*i*_) was detected by observations of first absence of mortality (pen shell alive at *p*_*i*_*t*_*0*_) and then indications of mortality by probable infection (pen shell dead or ill at *p*_*i*_*t*_*f*_). Given the available dataset, one backward simulation (from 2016/10/24 to 2016/09/09 in the Balearic Islands) and two forward simulations (from 2017/06/09 to 2017/07/24 in the L’Ametlla de Mar area and from 2017/10/27 to 2017/12/11 in North Catalonia) were produced in order to analyse the time for a particle to drift: (*i*) from *p*_1_ (visible mortality at *p*_1_*t*_0_) to *p*_2_ (no infection observed at *p*_2_*t*_0_ and mortality at *p*_2_*t*_*f*_) in the case of forward simulations (tracking particles destination), and inversely, (*ii*) from *p*_2_*t*_*f*_ to *p*_1_ in the case of backward simulation (tracking particles provenance). To predict the beginning of a local mortality event at a given point (*p*_1_ or *p*_2_ in the case of backward or forward simulation, respectively), the subset of simulated particles drifting within a buffer zone of 650 km^2^ around selected point (as center of such buffer zone) were selected before extracting the final time (arrival time; *t*_*f*_) of each of these particles. These predicted times were compared checking the time delay between empirical dates of *P. nobilis* infected in the starting (*p*_1_) and ending locations (*p*_2_).

The relationship with establishment success of the disease after arrival and environmental variables was addressed using a generalized additive model (GAM) with the *gam* function of the *mgcv* library within R software^74^. GAMs extend general linear models allowing for complex correlations between response and explanatory variables^75^. In this sense, the potential effect of temperature (*Temp*) and salinity (*Sal*) on the presence/absence of the disease (*Disease*) was tested fitting a binomial GAM [*Disease*~ s(*Temp*) + s(*Sal*)]. It is important to highlight that a subset of data was selected to fit the GAM (*n* = 271 observations; 232 presence and 39 absence of disease). Only data with a probable spatio-temporal prevalence of the disease were selected, avoiding the bias caused by the spatio-temporal absence of the disease (e.g., data from Eastern Mediterranean where the disease has not yet been detected and the *P. nobilis* populations are still healthy).

## Supplementary information

Simulation of disease tracking

Supplementary Figures 1A-B

Supplementary Dataset 1

## References

[1] Vicente N (1990). Estudio ecológico y protección del molusco lamelibranquio *Pinna nobilis* L., 1758 en la costa mediterránea. Iberus.

[2] Zavodnik, D., Hrs-Brenko, M. & Legac, M. Synopsis on the fan shell *Pinna nobilis* L. In the eastern Adriatic Sea. in *Les Espècies Marines à protéger en* Méditerranée (eds Boudouresque, C. F., Avon, M. & Gravez, V.) 169–178 (GIS Posidonie publications, 1991).

[3] Schulz P, Huber M (2013). Revision of the Worldwide Recent Pinnidae and some Remarks on Fossil European Pinnidae. Acta Conchyl..

[4] García-March JR, Garcia-Carrascosa AM, Peña AL (2002). *In Situ* Measurement of *Pinna nobilis* Shells for Age and Growth Studies: A New Device. Mar. Ecol..

[5] Katsanevakis S (2007). Growth and mortality rates of the fan mussel *Pinna nobilis* in Lake Vouliagmeni (Korinthiakos Gulf, Greece): A generalized additive modelling approach. Mar. Biol..

[6] Kersting DK, García-March JR (2017). Long-term assessment of recruitment, early stages and population dynamics of the endangered Mediterranean fan mussel *Pinna nobilis* in the Columbretes Islands (NW Mediterranean). Mar. Environ. Res..

[7] Rouanet E, Trigos S, Vicente N (2015). From youth to death of old age: the 50-year story of a *Pinna nobilis* fan mussel population at Port-Cros Island (Port-Cros National Park, Provence, Mediterranean Sea). Sci. Reports Port-Cros Natl. Park.

[8] Trigos S, García-March JR, Vicente N, Tena J, Torres J (2014). Utilization of muddy detritus as organic matter source by the fan mussel *Pinna nobilis*. Mediterr. Mar. Sci..

[9] Corriero G, Pronzato R (1987). Epibiontic sponges on the bivalve *Pinna nobilis*. Mar. Ecol. Prog. Ser..

[10] Giacobbe S (2002). Epibiontic mollusc communities on *Pinna nobilis* L. (Bivalvia, Mollusca). J. Nat. Hist..

[11] Addis P (2009). Density, size structure, shell orientation and epibiontic colonization of the fan mussel *Pinna nobilis* L. 1758 (Mollusca: Bivalvia) in three contrasting habitats in an estuarine area of Sardinia (W Mediterranean). Sci. Mar..

[12] Katsanevakis S (2016). Transplantation as a conservation action to protect the Mediterranean fan mussel *Pinna nobilis*. Mar. Ecol. Prog. Ser..

[13] Fiorito G, Gherardi F (1999). Prey-handling behaviour of *Octopus vulgaris* (Mollusca, Cephalopoda) on Bivalve preys. Behav. Processes.

[14] García-March JR, García-Carrascosa AM, Peña Cantero AL, Wang YG (2007). Population structure, mortality and growth of *Pinna nobilis* Linnaeus, 1758 (Mollusca, Bivalvia) at different depths in Moraira bay (Alicante, Western Mediterranean). Mar. Biol..

[15] Rabaoui L, Zouari ST, Hassine OK (2008). Ben. Two species of Crustacea (Decapoda) associated with the fan mussel, *Pinna nobilis* Linnaeus, 1758 (Mollusca, Bivalvia). Crustaceana.

[16] Sureda A, Natalotto A, Álvarez E, Deudero S (2013). Increased antioxidant response and capability to produce ROS in hemocytes of *Pinna nobilis* L. exposed to anthropogenic activity. Environ. Pollut..

[17] Alomar C, Vázquez-Luis M, Magraner K, Lozano L, Deudero S (2015). Evaluating stable isotopic signals in bivalve *Pinna nobilis* under different human pressures. J. Exp. Mar. Biol. Ecol..

[18] Vázquez-Luis M, Borg JA, Morell C, Banach-Esteve G, Deudero S (2015). Influence of boat anchoring on *Pinna nobilis*: A field experiment using mimic units. Mar. Freshw. Res..

[19] Hendriks IE (2013). Boat anchoring impacts coastal populations of the pen shell, The largest bivalve in the Mediterranean. Biol. Conserv..

[20] Katsanevakis S (2009). Population dynamics of the endangered fan mussel *Pinna nobilis* in a marine lake: A metapopulation matrix modeling approach. Mar. Biol..

[21] Basso L (2015). The Pen Shell, *Pinna nobilis*: A Review of Population Status and Recommended Research Priorities in the Mediterranean Sea. Adv. Mar. Biol..

[22] Deudero S, Vázquez-Luis M, Álvarez E (2015). Human stressors are driving coastal benthic long-lived sessile fan mussel *Pinna nobilis* population structure more than environmental stressors. PLoS ONE.

[23] Basso L, Hendriks IE, Duarte CM (2015). Juvenile Pen Shells (*Pinna nobilis*) tolerate acidification but are vulnerable to warming. Estuaries and Coasts.

[24] Fey SB (2015). Recent shifts in the occurrence, cause, and magnitude of animal mass mortality events. Proc. Natl. Acad. Sci..

[25] Langangen Ø (2017). Cascading effects of mass mortality events in Arctic marine communities. Glob. Chang. Biol..

[26] Vázquez-Luis M (2017). S.O.S. *Pinna nobilis*: A Mass Mortality Event in Western Mediterranean Sea. Front. Mar. Sci..

[27] Catanese G (2018). *Haplosporidium pinnae* sp. nov., a haplosporidan parasite associated with mass mortalities of the fan mussel, *Pinna nobilis*, in the Western Mediterranean Sea. J. Invertebr. Pathol..

[28] Katsanevakis Stelios (2019). The cryptogenic parasite Haplosporidium pinnae invades the Aegean Sea and causes the collapse of Pinna nobilis populations. Aquatic Invasions.

[29] Carella F (2019). A mycobacterial disease is associated with the silent mass mortality of the pen shell *Pinna nobilis* along the Tyrrhenian coastline of Italy. Sci. Rep..

[30] Panarese R (2019). *Haplosporidium pinnae* associated with mass mortality in endangered *Pinna nobilis* (Linnaeus 1758) fan mussels. J. Invertebr. Pathol..

[31] Darriba S (2017). First haplosporidan parasite reported infecting a member of the Superfamily Pinnoidea (*Pinna nobilis*) during a mortality event in Alicante (Spain, Western Mediterranean). J. Invertebr. Pathol..

[32] Ford SE, Haskin HH (1982). History and epizootiology of *Haplosporidium nelsoni* (MSX), an oyster pathogen in Delaware Bay, 1957-1980. J. Invertebr. Pathol..

[33] Haskin HH, Andrews JD (1988). Uncertainties and speculations about the life cycle of the eastern oyster pathogen *Haplosporidium nelsoni* (MSX). Dis. Process. Mar. Bivalve Molluscs.

[34] Hudson EB, Hill BJ (1991). Impact and spread of bonamiasis in the UK. Aquaculture.

[35] Laing I, Dunn P, Peeler EJ, Feist SW, Longshaw M (2014). Epidemiology of Bonamia in the UK, 1982 to 2012. Dis. Aquat. Organ..

[36] Hofmann E, Ford S, Powell E, Klinck J (2001). Modeling studies of the effect of climate variability on MSX disease in eastern oyster (*Crassostrea virginica*) populations. Hydrobiologia.

[37] Burreson EM, Ford SE (2004). A review of recent information on the Haplosporidia, with special reference to *Haplosporidium nelsoni* (MSX disease). Aquat. Living Resour..

[38] Ford SE, Haskin HH (1988). Comparison of *in vitro* salinity tolerance of the oyster parasite, *Haplosporidium nelsoni* (msx) and hemocytes from the host, *Crassostrea virginica*. Comp. Biochem. Physiol.–Part A Physiol..

[39] Andrews JD (1982). Epizootiology of late summer and fall infections of oysters by *Haplosporidium nelsoni*, and comparison to annual life cycle of *Haplosporidium costalis*, a typical haplosporidan. J. Shellfish Res..

[40] Hine PM (2002). Severe apicomplexan infection in the oyster *Ostrea chilensis*: A possible predisposing factor in bonamiosis. Dis. Aquat. Organ..

[41] Audemard C, Carnegie RB, Hill KM, Peterson CH, Burreson EM (2014). *Bonamia exitiosa* transmission among, and incidence in, Asian oyster *Crassostrea ariakensis* under warm euhaline conditions. Dis. Aquat. Organ..

[42] Arzul I (2009). Effects of temperature and salinity on the survival of *Bonamia ostreae*, a parasite infecting flat oysters *Ostrea edulis*. Dis. Aquat. Organ..

[43] Arzul, I. *et al*. *Bonamia ostreae* and *Ostrea edulis*: a stable host-parasite system in France? *XI International Symposium for Veterinary Epidemiology and Economics*, Cairns, Queensland, Australia, 6–11 August 2006. (2006).

[44] Arzul I, Carnegie RB (2015). New perspective on the haplosporidian parasites of molluscs. J. Invertebr. Pathol..

[45] Levin SA (1992). The problem of pattern and scale in ecology. Ecology.

[46] Palmer JRB (2017). Citizen science provides a reliable and scalable tool to track disease-carrying mosquitoes. Nat. Commun..

[47] Kroeck MA, Semenas L, Morsan EM (2008). Epidemiological study of *Bonamia* sp. in the native flat oyster, *Ostrea puelchana* from San Matías Gulf (NW Patagonia, Argentina). Aquaculture.

[48] Ford SE, Allam B, Xu Z (2009). Using bivalves as particle collectors with PCR detection to investigate the environmental distribution of *Haplosporidium nelsoni*. Dis. Aquat. Organ..

[49] Barber RD, Ford SE (1992). Occurrence and significance of ingested haplosporidan spores in the eastern oyster, *Crassostrea virginica* (Gmelin, 1791). J. Shellfish Res..

[50] Messerman NA, Bowden TJ (2016). Survey of potential reservoir species for the oyster parasite Multinucleate Sphere X (*Haplosporidium nelsoni*) in and around oyster farms in the Damariscotta River Estuary, Maine. J. Shellfish Res..

[51] Powell EN, Klinck JM, Ford SE, Hofmann EE, Jordan SJ (1999). Modeling the MSX parasite in eastern oyster (*Crassostrea virginica*) populations. III. Regional application and the problem of transmission. J. Shellfish Res..

[52] Vidal-Vijande E, Pascual A, Barnier B, Molines J-M, Tintoré J (2011). Analysis of a 44-year hindcast for the Mediterranean Sea: comparison with altimetry and *in situ* observations. Sci. Mar..

[53] Prado P, Caiola N, Ibáñez C (2014). Habitat use by a large population of *Pinna nobilis* in shallow waters. Sci. Mar..

[54] Salat J, Cruzado A (1981). Masses d’eau dans la Méditerranée occidentale: Mer Catalane et eaux adjacentes. Rapp. Comm. Int. pour l’exploration Sci. la mer Méditerranée.

[55] Pérez-Ruzafa, Á., Marcos, C. & Gilabert, J. The ecology of the Mar Menor Coastal Lagoon: a fast changing ecosystem under human pressure. In *Coastal lagoons. Ecosystem processes and modeling for sustainable use and development* (eds Gönenç, I. E. & Wolflin, J. P.) 392–422 (CRC Press Boca Raton, 2005).

[56] Vargas-Yáñez M (2017). Updating temperature and salinity mean values and trends in the Western Mediterranean: The RADMED project. Prog. Oceanogr..

[57] Dickinson JL (2012). The current state of citizen science as a tool for ecological research and public engagement. Front. Ecol. Environ..

[58] Kosmala M, Wiggins A, Swanson A, Simmons B (2016). Assessing data quality in citizen science. Front. Ecol. Environ..

[59] Wiggins A, Newman G, Stevenson RD, Crowston K (2011). Mechanisms for data quality and validation in citizen science. Proceedings - 7th IEEE International Conference on e-Science Workshops, eScienceW.

[60] Nebot-Colomer E, Vázquez-Luis M, Gracía-March JR, Deudero S (2016). Population Structure and Growth of the Threatened Pen Shell, *Pinna rudis* (Linnaeus, 1758) in a Western Mediterranean Marine Protected Area. Mediterr. Mar. Sci..

[61] Zenetos A (2012). Alien species in the Mediterranean Sea by 2012. A contribution to the application of European Union’s Marine Strategy Framework Directive (MSFD). Part 2. Introduction trends and pathways. Mediterr. Mar. Sci..

[62] Wesselmann M (2018). Genetic and oceanographic tools reveal high population connectivity and diversity in the endangered pen shell *Pinna nobilis*. Sci. Rep..

[63] Lande R (1988). Genetics and demography in biological conservation. Science.

[64] González-Wangüemert M (2018). Gene pool and connectivity patterns of *Pinna nobilis* in the Balearic Islands (Spain, Western Mediterranean Sea): Implications for its conservation through restocking. Aquat. Conserv. Mar. Freshw. Ecosyst..

[65] Trigos, S. Estudio de la ecofisiología y ensayo de cultivio de la nacra *Pinna nobilis* Linnaeus, 1758. (Universidad Católica de Valencia, 2017).

[66] Katsanevakis S (2011). ‘Protected’ marine shelled molluscs: Thriving in Greek seafood restaurants. Mediterr. Mar. Sci..

[67] Vázquez-Luis M (2017). Situación actual de *Pinna nobilis* en España. Nuevos datos de mortandad, individuos vivos y captación de juveniles. Not. SEM.

[68] La SEM (2017). nacra *Pinna nobilis*. Not. SEM.

[69] Good SA, Martin MJ, Rayner NA (2013). EN4: Quality controlled ocean temperature and salinity profiles and monthly objective analyses with uncertainty estimates. J. Geophys. Res. Ocean..

[70] Juza M (2016). SOCIB operational ocean forecasting system and multi-platform validation in the Western Mediterranean Sea. J. Oper. Oceanogr..

[71] Mourre, B. *et al*. Assessment of high-resolution regional ocean prediction systems using multi-platform observations: illustrations in the Western Mediterranean Sea. In *New Frontiers in* Operationl *Oceanography* (eds Chassignet, E., Verron, J., Pascual, A. & Tintoré, J.) (2018).

[72] Shchepetkin AF, McWilliams JC (2005). The regional oceanic modeling system (ROMS): a split-explicit, free-surface, topography-following-coordinate oceanic model. Ocean Model..

[73] Döös Kristofer, Kjellsson Joakim, Jönsson Bror (2013). TRACMASS—A Lagrangian Trajectory Model. Preventive Methods for Coastal Protection.

[74] Wood, S. N. Generalized Additive Models: An Introduction with R. *Chapman& Hall/CRC, Boca Rat*. 391 pp., 10.1111/j.1541-0420.2007.00905_3.x (2006).

[75] Hastie T, Tibshirani R (1986). Generalized Additive Models. Stat. Sci..

