# Supplementary material for: Tracking a mass mortality outbreak of pen shell Pinna nobilis populations: A collaborative effort of scientists and citizens
Source: Sci Rep. 2019 Sep 16;9:13355. doi: 10.1038/s41598-019-49808-4 (PMC6746856; doi:10.1038/s41598-019-49808-4)
Supplement: Supplementary file 2 — Supplementary Figures 1A-B [file 41598_2019_49808_MOESM2_ESM.pdf]

Supplementary Figures for

“Tracking a mass mortality outbreak of pen shell *Pinna nobilis* populations: A collaborative effort of scientists and citizens”

Miguel Cabanellas-Reboredo, Maite Vázquez-Luis, Baptiste Mourre, Elvira Álvarez, Salud Deudero, Ángel Amores, Piero Addis, Enric Ballesteros, Agustín Barrajón, Stefania Coppa, José Rafael García-March, Salvatore Giacobbe, Francisca Giménez Casalduero, Louis Hadjioannou, Santiago V. Jiménez-Gutiérrez, Stelios Katsanevakis, Diego Kersting, Vesna Mačić, Borut Mavrič, Francesco Paolo Patti, Serge Planes, Patricia Prado, Jordi Sánchez, José Tena-Medialdea, Jean de Vaugelas, Nardo Vicente, Fatima Zohra Belkhamssa, Ivan Zupan, and Iris E. Hendriks

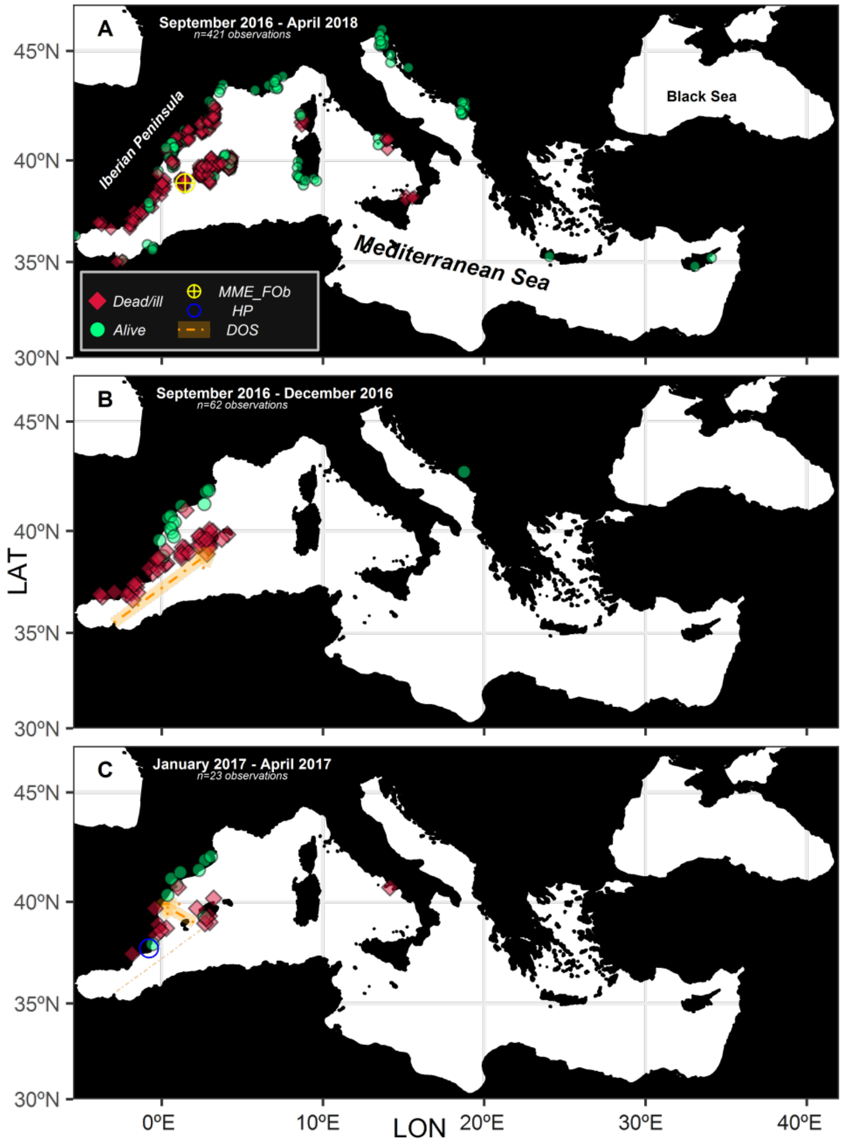

Figure 1A. Observations of the healthy status of *P. nobilis* (Dead/ill *vs.* Alive): A) the whole period from September 2016 to April 2018, B) September/December 2016 and C) January/April 2017. Note that the crossed yellow point indicates the zone where the mortality was observed for the first time (Mass Mortality Event First Observation; *MME_FOb*), blue empty circles surround Healthy Populations (*HP*) and orange arrows denote Disease Observations Sequence (DOS).

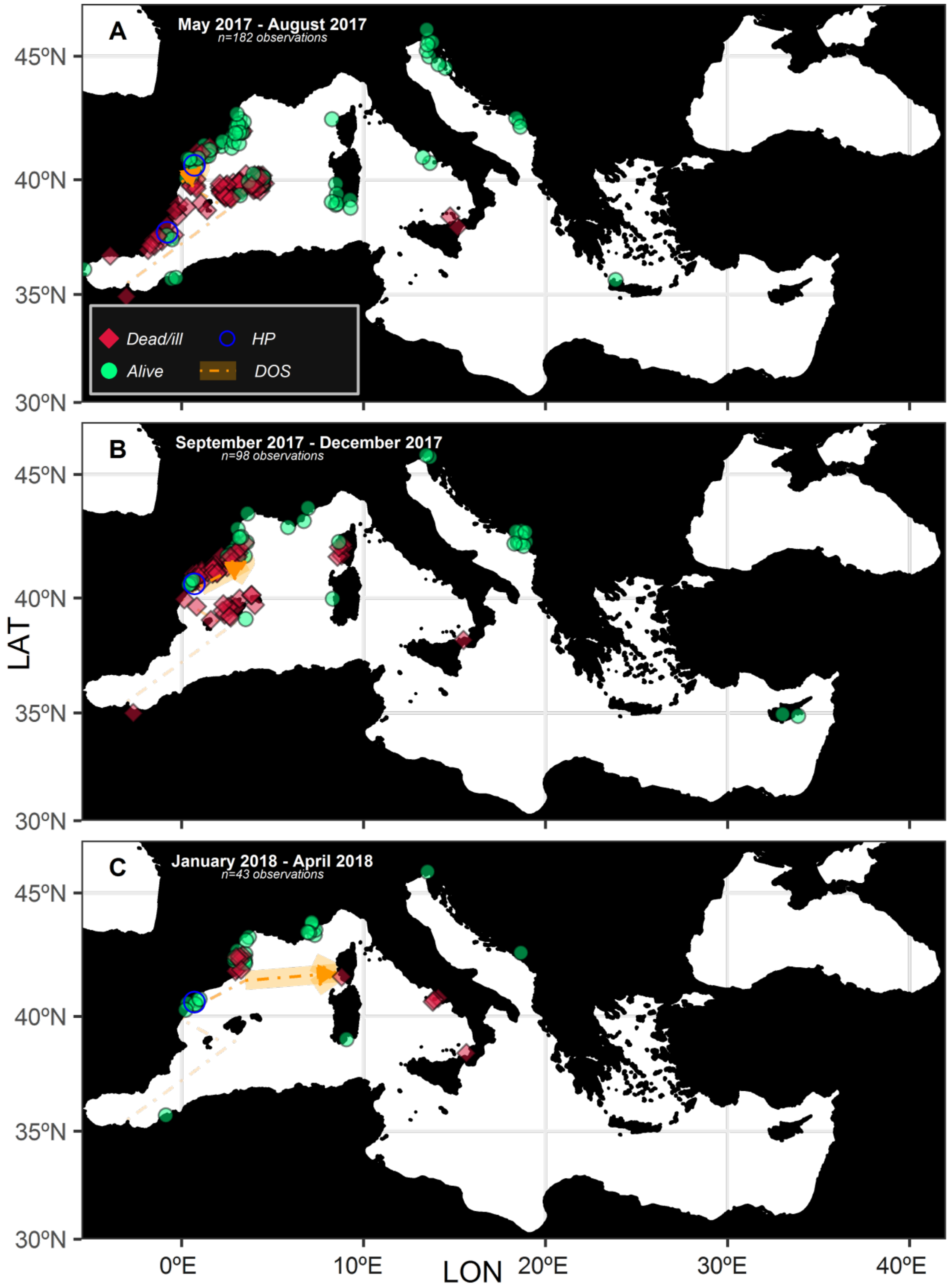

Figure 1B. Observations of the healthy status of *P. nobilis* (Dead/ill *vs*. Alive): A) period from May to August 2017, E) September/December 2017 and F) January/April 2018. Blue empty circles indicate surround Healthy Populations (HP) and orange arrows denote Disease observations sequence (DOS).
